# Integration of Long Non-Coding RNA and mRNA Profiling Reveals the Mechanisms of Different Dietary NFC/NDF Ratios Induced Rumen Development in Calves

**DOI:** 10.3390/ani12050650

**Published:** 2022-03-03

**Authors:** Jichao Li, Mingming Xue, Liyang Zhang, Lanjie Li, Hongxia Lian, Ming Li, Tengyun Gao, Tong Fu, Yan Tu

**Affiliations:** 1 Henan International Joint Laboratory of Nutrition Regulation and Ecological Raising of Domestic Animal, College of Animal Science and Technology, Henan Agricultural University, Zhengzhou 450046, China; ljcsci@126.com (J.L.); 15736780320@163.com (M.X.); zhangliyang@henau.edu.cn (L.Z.); lhx263@sina.com (H.L.); 13803849306@163.com (M.L.); dairycow@163.com (T.G.); 2 Key Laboratory of Feed Biotechnology of Ministry of Agriculture, Feed Research Institute, Chinese Academy of Agricultural Sciences, Beijing 100081, China; lilanjie92@163.com

**Keywords:** calves, long non-coding RNA, mRNA, non-fibrous carbohydrate to neutral detergent fiber, rumen development

## Abstract

**Simple Summary:**

Altering carbohydrate source and form affects the development of the rumen epithelium. Calves fed diets with one of three different ratios of non-fibrous carbohydrate to neutral detergent fiber (NFC/NDF) was conducted to reveal putative mechanisms and pathways affected by and responsible for rumen development. Calves in the high NFC/NDF ratio (1.10) group had higher average daily gain, and ruminal papillae developed flatter than calves in the low NFC/NDF ratio (0.60) group. Transcriptomics identified that a large number of differentially expressed genes were significantly enriched in pathways related to rumen epithelia development including focal adhesion, Wnt signaling pathway, thyroid hormone signaling pathway, regulation of actin cytoskeleton and cGMP-PKG signaling pathway. The lncRNA-mRNA network analysis revealed that some target genes including XLOC_068691 and monoamine oxidase B (MOAB), XLOC_023657 and dickkopf homolog 2 (DKK2), XLOC_064331 and protein phosphatase 1 regulatory subunit 12A (PPP1R12A) were affected by the NCF/NDF ratios fed. The pathways and affected genes may serve as markers for the further investigation into the mechanisms regulating dietary and luminal factors impacting rumen development in growing ruminants.

**Abstract:**

The aim of the present study was to explore the effects of dietary non-fibrous carbohydrate to neutral detergent fiber (NFC/NDF) ratios on rumen development of calves, and to investigate the mechanisms by integrating of lncRNA and mRNA profiling. Forty-five weaned Charolais hybrid calves [body weight = 94.38 ± 2.50 kg; age = 70 ± 2.69 d] were randomly assigned to 1 of 3 treatment groups with different dietary NFC/NDF ratios: 1.10 (H group), 0.94 (M group) and 0.60 (L group), respectively. The ventral sac of the rumen was sampled for morphological observation and transcriptional sequencing. The average daily gain of calves in the high NFC/NDF ratio group was significantly higher than that in other groups (*p* < 0.05). Papillae width was largest in high NFC/NDF ratio group calves (*p* < 0.05). Identified differentially expressed genes that were significantly enriched in pathways closely related to rumen epithelial development included focal adhesion, Wingless-int signaling pathway, thyroid hormone signaling pathway, regulation of actin cytoskeleton and cGMP-PKG signaling pathway. The lncRNA-mRNA network included XLOC_068691 and MOAB, XLOC_023657 and DKK2, XLOC_064331 and PPP1R12A which we interpret to mean they have important regulatory roles in calve rumen development. These findings will serve as a theoretical basis for further analysis of the molecular genetic mechanism of dietary factors affecting rumen development in calves.

## 1. Introduction

A fully functional rumen is critical for ration digestion, nutrient absorption in support of metabolism in maturing ruminants. The development of the rumen affects the postnatal growth of calves directly [1]. While rumen development is a gradual process, obvious changes in weight and volume of the rumen occur upon introduction of solid feed to the ration. Calf age, composition and physical form of the ration, ruminal volatile fatty acid (VFA) concentrations, pH and microbial populations have all been demonstrated to affect the morphological development of the rumen [2,3,4]. The feed composition and physical form are external factors. The fluctuation of rumen pH is an internal cause. The difference in the composition of volatile fatty acids is the causal factor [5]. There has been extensive research demonstrating that different carbohydrate sources and dietary forms affect the development of rumen epithelial tissue and muscle thickness of calves. The high neutral detergent fiber (NDF) diets decreased the incidence of coalescing rumen papillae, and increased the muscle thickness, which had a better promotive effect on rumen development among different carbohydrate treatments [6]. Increasing NDF in the ration could ameliorate harsh rumen environments (low pH) associated with high concentrate rations [7]. Concentrate diet supplementation with alfalfa hay has been demonstrated to reduce the formation of rumen wall plaques and improve the macroscopic and microscopic appearance of the rumen [8]. However, butyrate, produced from the fermentation of concentrate, plays an important role in stimulating the development of rumen mucosa and is more effective in inducing development than roughage feeding alone [9]. The appropriate non-fibrous carbohydrate to neutral detergent fiber (NFC/NDF) ratio for rumen development of calves is still the focus of current research.

The long non-coding RNAs (lncRNAs) play an important role in finely modulating the mechanisms of many biological activities, such as epigenetic regulation, cell cycle regulation and cell differentiation [10,11,12]. Further study from the perspective of lncRNA would be conducive to explore the molecular mechanisms of dietary factors affecting calves’ rumen development. Wang et al. (2016) used RNA-seq analysis to reveal 225 differentially expressed genes between the rumen of milk-fed and feed-fed lambs, providing insights into rumen development [13]. Ibeagha-Awemu et al. (2018) detected 4243 (88 known and 4155 novel) lncRNAs in rumen tissues and analyzed mechanisms regulating the development of the rumen in calves during the early weeks of life [14]. Sla et al. (2020) established transcriptomic analyses of cattle rumen epithelial primary cells culture to elucidate interactions between butyrate and rumen development [15]. While there is a great deal of speculation regarding the mechanisms regulating rumen development, a complete description of the mechanisms has not been delineated conclusively. The specific mechanism of NFC/NDF ratios in dietary composition affecting rumen development needs to be further clarified.

We hypothesized that lncRNA played an important role in regulating the rumen development of calves fed a diet with different carbohydrate compositions. Therefore, we conducted a feeding trial with a range of NCF/NDF ratios in the rations fed to calves and used lncRNA and mRNA profiling to elucidate putative pathways and molecules regulating the process in order to establish putative mechanisms involved.

## 2. Materials and Methods

### 2.1. Animals and Experimental Design

Forty-five Charolais hybrid bull calves [body weight (BW) = 94.38 ± 2.50 kg; age = 70 ± 2.69 d] were randomly assigned to 1 of 3 treatment groups with different dietary NFC/NDF ratios: 1.10 (H group), 0.94 (M group) and 0.60 (L group), respectively. Calves were fed with the total mixed ration (TMR). The TMR were formulated to meet the nutritional requirements of calves with 150 kg body weight and 1.0 kg/d daily gain according to the Beef Cattle Raising Standard of China (2004) [16]. Calves were fed in separate pens in a calf hutch (4.7 m × 1.5 m). The animals were given a 15-day adaptation period followed by a 90-day feeding experiment. Calves were allowed ad libitum access to experimental diets and water throughout the feeding trial. The ingredients and chemical composition of diets are shown in Table 1.

Three calves from each experimental group were randomly chosen and sacrificed. Rumen fluid samples were filtered with gauze and collected to determine pH and volatile fatty acid. The ventral sac of the rumen was collected for subsequent analysis, and the site with the highest capillary blood flow per unit weight mucosa of any location within the rumen was sampled [17]. Three pieces of the fresh rumen samples from each calf were preserved for rumen sections. Meanwhile, whole-thickness tissue samples from the rumen were collected with aluminum foil, and then immediately snap-frozen in liquid nitrogen, and stored at −80 °C until RNA extraction.

### 2.2. Feed Intake and Growth Performance

The ort of each calf was collected before morning feeding to calculate dry matter intake (DMI). The initial body weight of calves was recorded and subsequently weighed each 30 d thereafter during the experiment to calculate the average daily gain (ADG).

### 2.3. Preparation and Observation of Rumen Sections

Externalization and isolation of rumen samples was conducted as described in the instructions [18]. The 2 cm × 2 cm sections of rumen tissue were cut with sterile surgical scissors, washed in PBS buffer (pH = 7.2) and fixed in 4% paraformaldehyde. The rumen tissue was then dehydrated, cleared and embedded in paraffin. The 6 μm sections were stained with hematoxylin-eosin (H&E) after the sample was segmented. The papillae length, width and muscle layer thickness were measured 5 times using the Motic images advanced v3.2.

### 2.4. RNA Extraction, Library Construction, and Sequencing

The H group and L group rumen samples of calves with great differences from morphological observation were selected for transcriptional sequencing. Total RNA was extracted from the calves’ rumen samples using a Trizol Reagent (Invitrogen, Carlsbad, CA, USA) according to the manufacturer’s protocol. The extracted RNA was quantified, and the integrity was assessed. The total RNA content of each sample was 3 μg, which was used as the input material of RNA sample. Subsequently, sequencing libraries were generated with NEBNext^®^ Ultra™ (NEB, Ipswich, MA, USA). The quality evaluation of library by using the Agilent Bioanalyzer 2100 system (Agilent Technologies, Santa Clara, CA, USA). The Illumina Hiseq 2500 platform was used to sequence the libraries (Beijing, China).

Raw data in fastq format was processed by internal Perl scripts. The contents of Q20, Q30 and GC about the clean data obtained were calculated. Further analysis was carried out following high quality clean data.

### 2.5. Data Mapping and Transcriptome Assembly

Index of the reference genome was built using Bowtie v 2.0.6, and the filtered reads were mapped to the bovine reference genome (ftp://ftp.ensembl.org/pub/release-80/fasta/bos_taurus/dna/ (accessed on 20 February 2019)) using TopHat v2.0.9. The mapped reads were assembled and transcripts were constructed using the Cufflinks software [19]. The Cufflinks transcript has specific parameters for the strand-specific library to provide accurate transcript chain orientation information.

### 2.6. LncRNA Prediction

The coding ability of potential lncRNAs was estimated with the Coding Potential Calculator (CPC), Coding-Non-Coding Index (CNCI) [20], Pfam Scan (v1.3) and Phylogenetic codon substitution frequency (PhyloCSF). The filter criteria of lncRNAs were as follows: (1) exon count ≥2; (2) transcript length >200 nt; (3) transcripts were screened by Cuffcompare software, and lncRNAs that overlapped with the exon region of the spliced transcripts in the database were used as lncRNAs annotated in the database. (4) Cuffquant was selected to calculate transcripts with expected number of Fragments Per Kilobase of transcript sequence per Millions base pairs sequenced (FPKM) ≥0.5. The lncRNA data set predicted in this analysis was an intersection of transcripts without coding potential, while transcripts with uncertain coding potential (TUCP) were screened for transcripts with coding potential identified by at least one coding potential prediction software.

### 2.7. Structural and Conservative Analysis

The comparison of lncRNA and mRNA transcript length, exon number, open reading frame (ORF) length and sequence conservation were observed. The ORF sequence of the mRNA was extracted by known gene structure annotation, and the ORF sequence of lncRNA was predicted by EMBOSS. The phyloFit was used to compute phylogenetic models for conserved and non-conserved regions among species and then gave the model and HMM transition parameters to phyloP to compute a set of conservation scores of lncRNA and coding genes.

### 2.8. Quantitation of Gene Expression

The expression of transcripts were expressed as FPKM values using the Cuffdiff (http://cole-trapnell-lab.github.io/cufflinks/cuffdiff/index.html (accessed on 18 March 2019)). Under specific experimental operations, the Pearson correlation coefficient (R^2^) is required to be ≥0.8. The Cuffdiff provided statistical routines for determining differential expression in digital transcript or gene expression data using a model based on the negative binomial distribution [21]. Transcripts with a *p*-adjust < 0.05 were assigned as differentially expressed. Differentially expressed lncRNAs (DE-lncRNAs) and mRNAs (DE-mRNAs) for comparison between two groups were identified with a q-value < 0.05 and |(fold change)| ≥ 4 as the cutoff points.

### 2.9. Prediction of lncRNA Targets

To classify lncRNAs cis-target genes, we searched coding genes 10 k/100 k upstream and downstream of DE-lncRNAs. To classify lncRNAs trans-target genes, we used the correlation analysis between lncRNAs and genes expression in samples to predict DE-lncRNAs target genes, and to analyze the correlation between lncRNAs and genes in samples by Pearson correlation coefficient method and identified genes with absolute correlation greater than 0.95 as DE-lncRNAs trans-target genes. The DE-lncRNAs and their corresponding differentially expressed (DE) cis- and trans-target genes were used to construct lncRNAs-genes interaction networks using the Cytoscape program.

### 2.10. Gene Ontology and Kyoto Encyclopedia of Genes and Genomes Analysis

Gene ontology (GO) enrichment analysis was performed for differentially expressed genes (DEGs) or lncRNA target genes by GOseq R package to correct gene length bias. The GO term with a *p* value less than 0.05 was identified to be significantly enriched in DEGs. Using KOBAS software testing KEGG pathways of DEGs or lncRNA statistical enrichment of the target genes (http://www.genome.jp/kegg/ (accessed on 25 March 2019)).

### 2.11. Quantitative Real-Time PCR Analysis

Primers were designed using Primer-BLAST on the NCBI website (http://www.ncbi.nlm.nih.gov/tools/ primer-blast/ (accessed on 15 April 2019)). The final concentration of each primer was 10 μmol/μL. The primers used for qRT-PCR are described in Table 2. Total RNA was extracted using TRIzol reagent, and then was reverse transcribed using the PrimeScript RT reagent Kit with gDNA Eraser (TaKaRa, Dalian, China) following the manufacturer’s instructions. All qRT-PCR procedures were performed in 3 independent biological replicates of each treatment, and each sample consisted of 3 technical replicates. Real-time PCR was carried out using the SYBR Premix Ex Taq II kit (TaKaRa, Dalian, China) on a LightCycler 96 instrument (Roche, Indianapolis, IN, USA). Cycling parameters were 95 °C for 5 min, followed by 40 cycles of 95 °C for 15 s, 60 °C for 30 s and 72 °C for 30 s. Melting curve analyses were performed following amplifications. The 2^−ΔΔCt^ method was used to determine the relative mRNA and lncRNA abundance [22].

### 2.12. Statistical Analysis

The data were analyzed using SAS/STAT software (version 9.2, SAS Institute Inc., Cary, NC, USA). The results of rumen slicing were analyzed using the ANOVA procedure. Statistically significant differences among treatments were assessed using Duncan’s test. The data of qRT-PCR were evaluated for differences between groups by Student’s *t* test. Data are presented as the mean ± standard error of the mean. Differences were considered significant at *p* < 0.05.

## 3. Results

### 3.1. Feed Intake and Growth Performance

The results of feed intake and growth performance are shown in Table 3. There was no significant difference in final body weight among groups (*p* > 0.05). The DMI and F/G were not affected by dietary composition (*p* > 0.05). However, the body weight gain and ADG of calves in H group was significantly higher than that in other groups (*p* < 0.05).

### 3.2. Rumen Fermentation Parameters and Morphological Observation of Rumen Tissue

**Figure 1:**
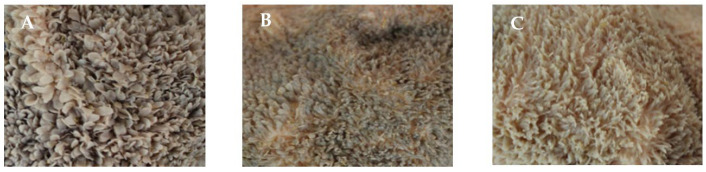
Observation of ruminal epithelial morphology. (**A**) H group (NFC/NDF = 1.10). (**B**) M group (NFC/NDF = 0.94). (**C**) L group (NFC/NDF = 0.60).

**Figure 2:**
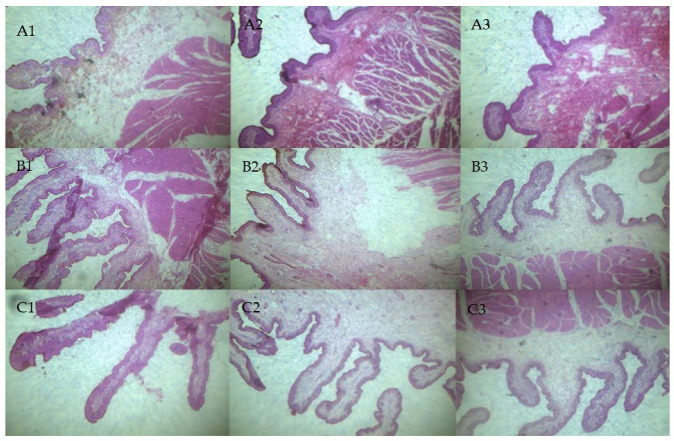
Histological observation of calf rumen tissues. (**A1–A3**) Rumen tissues of calves in H group. (**B1–B3**) Rumen tissues of calves in M group. (**C1–C3**) Rumen tissues of calves in L group.

The results of rumen fermentation parameters, rumen histological observation and sections are shown in Figure 1 and Figure 2 and Table 4. The acetate concentration of calves in H group was significantly lower than that in the other two groups (*p* < 0.05), and propionate concentration was significantly higher than that in L group (*p* < 0.05). No significant difference in butyrate concentration was observed among the groups (*p* > 0.05). From the observation results, rumen development was obviously different with treatments. The effects of dietary NFC/NDF ratios on the development of rumen papillae were mainly reflected in the papilla width and length. The rumen papillae length was longest in L group calves (*p* < 0.05), and papillae width was largest in H group calves (*p* < 0.05). The H group papillae width were 10.81% and 60.18% higher than that in M and L group, respectively. There was no significant difference in muscle layer thickness among the three groups of calves (*p* > 0.05).

### 3.3. High-Throughput Sequencing and Quality Control

Sequencing results are shown in Table 5. A total of 612,837,400 raw reads were produced from six cDNA libraries. After quality control, 597,793,954 clean reads were obtained. Overall, 82.96% to 85.10% of the clean reads were consistent with the Bos taurus reference genome. Splicing reads were segmented from 18.07% to 22.23% on two exons. Additionally, 57.47% to 64.54% of reads were mapped to non-spliced reads.

### 3.4. Identification of lncRNAs and mRNAs in Calves Rumen

**Figure 3:**
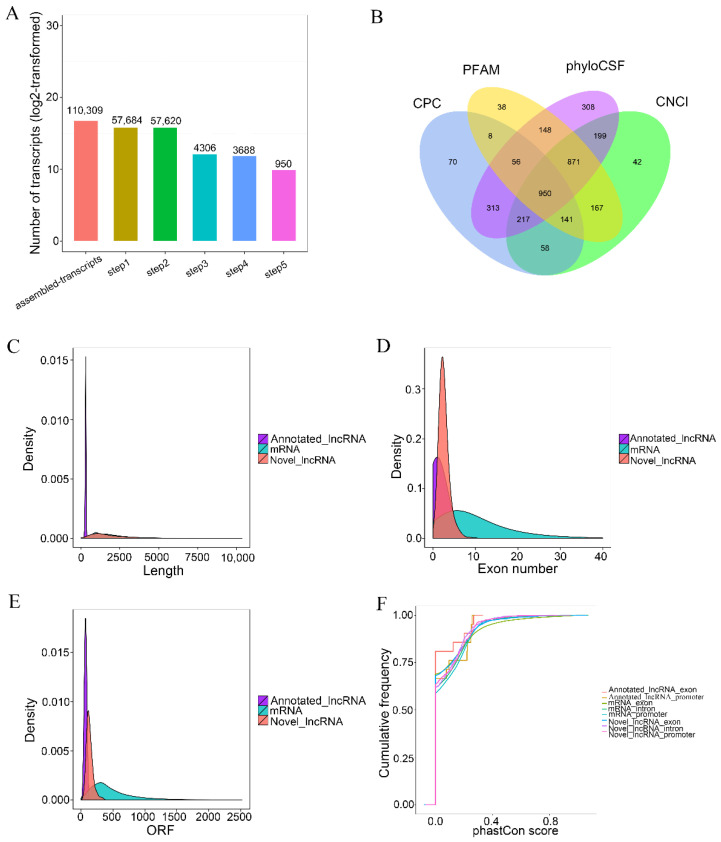
The features of calves’ rumen lncRNAs. (**A**) LncRNA screening statistics. The abscissa is the number of screening steps, and the ordinate is the number of transcripts after the screening of corresponding steps. (**B**) The screening results are shown in a Venn diagram. The sum of the numbers in each large circle represents the total number of noncoding transcripts of the software, and the overlapping part of the circle represents the common noncoding transcripts among the software. (**C**) Length comparison of LncRNA and mRNA. (**D**) Exon numbers per RNA. (**E**), lncRNA and mRNA ORF length density. (**F**) Conservation of RNA promoters and exons in calves’ rumen.

The number of transcripts screened by five steps are shown in Figure 3A. The noncoding transcripts identified by each software were counted and drawn into a Venn diagram to visually display the number of common and unique noncoding transcripts predicted by each method in Figure 3B, the transcripts length in Figure 3C, exon number in Figure 3D, ORF length in Figure 3E, and conservation of lncRNAs and mRNAs which were analyzed in Figure 3F. The results showed that 950 novel lncRNAs, 21 annotated lncRNAs and 22,094 mRNAs were obtained from six calve’s rumen samples. The length distribution pattern of the novel lncRNAs was similar to the mRNA that was significantly longer than the annotated lncRNAs. The exon number of the annotated lncRNAs was one, and the exon number of the novel lncRNAs was two to five. The ORF length of the lncRNAs mainly ranged from 40 to 200 bp. We found that the exon number of lncRNAs were lower than mRNAs, and their ORF length were shorter. The exons, introns and promoters of the newly identified lncRNAs in the rumen were less conserved than protein-encoding transcripts.

### 3.5. Differential Expression Analysis of lncRNAs and mRNAs

**Figure 4:**
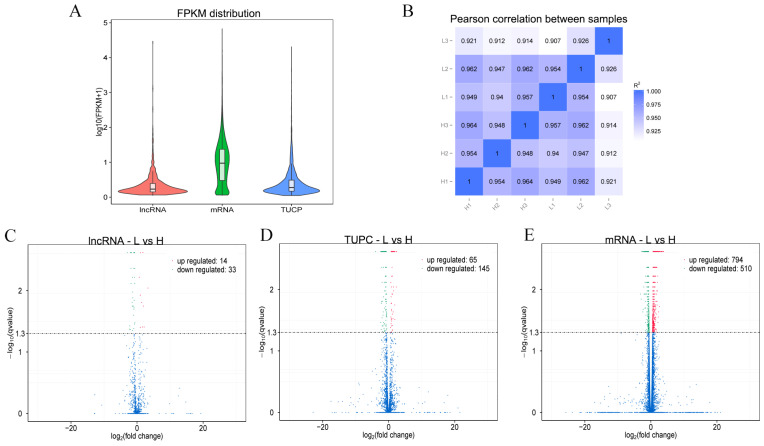
Comparative analysis of expression levels of different types of transcripts. (**A**) Overall expression level. (**B**) Correlation of gene expression levels among samples. (**C**) DE-lncRNA volcano plot between the two groups. (**D**) DE-TUCP volcano plot between the two groups. (**E**) DE-mRNA volcano plot between the two groups.

The expression levels of lncRNA, TUCP and mRNA were compared and analyzed to obtain the difference in the overall expression level of different types of transcripts, and the results are shown in Figure 4A. Expression correlation among samples was examined in Figure 4B. The volcano plots of differentially expressed transcripts are shown in Figure 4C–E. Correlation of gene expression levels between samples was used to attest experimental reliability and sample selection rationality. We compared the expression levels of transcript between high to low NFC/NDF ratio groups. Pearson correlation analysis showed that there was a high correlation among the samples in the groups, which indicated that the sampling was reasonable. Differential expression analysis of different types of transcripts (lncRNA, TUCP and mRNA) as a whole did not result in molecular type preference. There were 47 DE-lncRNAs (14 up-regulated and 33 down-regulated), 210 differentially expressed TUCPs (65 up-regulated and 145 down-regulated) and 1304 DE-mRNAs (794 up-regulated and 510 down-regulated) were identified based on the criteria of q-value < 0.05 and |(fold change)| ≥ 4 by comparisons of samples collected from rumen.

### 3.6. Target Gene Prediction and Functional Enrichment Analysis

**Figure 5:**
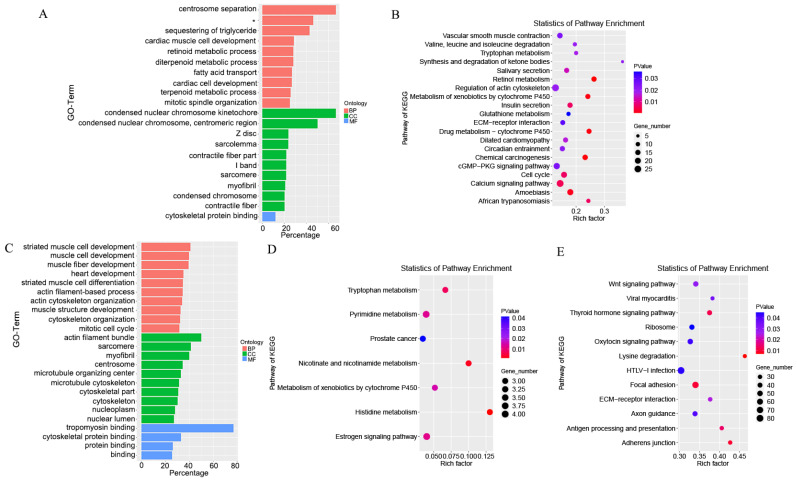
Functional enrichment analysis of differential expression RNAs. (**A**) GO categories enrichment analysis of DE-mRNAs. (**B**) KEGG pathways enrichment analysis of DE-mRNAs. (**C**) GO categories enrichment analysis of trans-target genes. (**D**) KEGG pathways enrichment analysis of trans-target genes. (**E**) KEGG pathways enrichment analysis of cis-target genes. * Regulation of ryanodine-sensitive calcium-release channel activity.

The biological functions of lncRNA were predicted through the co-location and co-expression with protein-coding genes. The DE-mRNAs and trans-target genes were analyzed using GO (Gene Ontology) categories enrichment in Figure 5A,C. The DE-mRNAs, trans-target genes and cis−target genes were analyzed with KEGG pathways enrichment in Figure 5B,D,E. A total of 4,499 cis-target genes for the 47 DE-lncRNAs were identified. A total of 99 trans-target genes for the 36 DE-lncRNAs were identified. The DEGs were mainly enriched in centrosome separation, mitotic spindle organization, condensed nuclear chromosome kinetochore, condensed nuclear chromosome, centromeric region, condensed chromosome and cytoskeletal protein binding. The significantly enriched pathways of DEGs included retinol metabolism, cell cycle, regulation of actin cytoskeleton, cGMP-PKG signaling pathway and extracellular matrix (ECM) receptor interaction. The cis-target genes of DE-lncRNAs were mainly enriched in actin filament-based process, actin cytoskeleton organization, mitotic cell cycle, centrosome, tropomyosin binding and cytoskeletal protein binding. The KEGG enrichment were mainly enriched in tryptophan metabolism, nicotinate and nicotinamide metabolism, histidine metabolism and estrogen signaling pathway. The cis-target genes of DE-lncRNAs GO enrichment were not significant. The significantly enriched pathways of cis-target genes included lysine degradation, thyroid hormone signaling pathway, Wingless-int (Wnt) signaling pathway, focal adhesion and adherens junction.

### 3.7. Integrated Analysis of Differentially Expressed lncRNAs and mRNA

**Figure 6:**
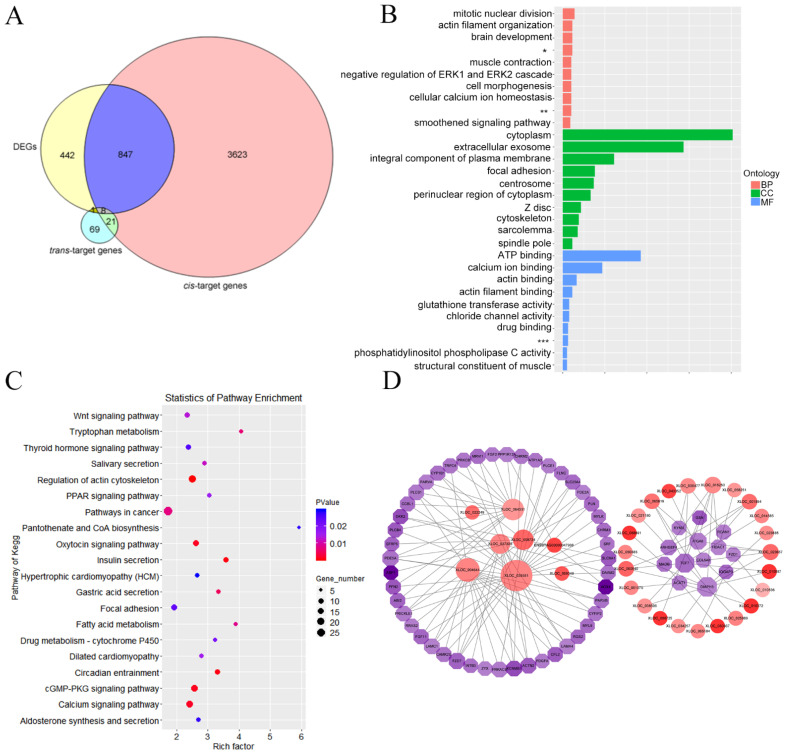
Functional enrichment analysis of differential expression target gene. (**A**) Venn diagram of differential expression cis- and trans-target genes. (**B**) GO categories enrichment analysis of DE-target genes. (**C**) KEGG pathways enrichment analysis of DE-target genes. (**D**) Differential gene interactions with pathways related to rumen development. * Negative regulation of canonical Wnt signaling pathway, ** Positive regulation of sequence-specific DNA binding transcription factor activity, *** Ligand-dependent nuclear receptor transcription coactivator activity.

The Venn diagram of differential expression cis- and trans-target genes is exhibited in Figure 6A. The DE-target genes were analyzed with GO categories enrichment in Figure 6B. The DE-target genes were analyzed with KEGG pathways enrichment in Figure 6C. Differential genes interactions with pathways related to rumen development were shown in Figure 6D. A total of 855 cis-target differentially expressed genes for the 47 DE-lncRNAs were identified. A total of nine differentially expressed trans-target genes for the eight DE-lncRNAs were identified. Among them, eight differentially expressed mRNAs were co-expressed and co-located by DE-lncRNAs. It was found that only the relationship between XLOC_064331 and protein phosphatase 1 regulatory subunit 12A (PPP1R12A), XLOC_004641 and Meis homeobox 2 (MEIS2), XLOC_068691 and monoamine oxidase B (MAOB), XLOC_061079 and ankyrin repeat domain 24 (ANKRD24) were not only co-expressed but also co-located. The differentially expressed cis-target genes of DE-lncRNAs were mainly enriched in mitotic nuclear division, actin filament organization, negative regulation of canonical Wnt signaling pathway, integral component of plasma membrane, centrosome and actin binding. The significantly enriched pathways included focal adhesion, Wnt signaling pathway, thyroid hormone signaling pathway, regulation of actin cytoskeleton and cGMP-PKG signaling pathway. To further investigate the function of the intersecting genes in rumen development, we visualized the integrated lncRNA-mRNA networking among DE-lncRNAs and their target genes significantly enriched in the above-mentioned pathways. The results showed that a large number of DEGs were related to organism growth and development including aldehyde oxidase 4 (AOX4), amine oxidase, copper containing 1 (AOC1), potassium calcium-activated channel subfamily M regulatory beta subunit 1 (KCNMB1), dickkopf 2 (DKK2), transcription factor 7 (TCF7), diaphanous-related formin-3 (DIAPH3), collagen type V alpha 3 chain (COL5A3), and gelsolin (GSN), and were potentially targeted by ENSBTAG00000047908, XLOC_008734, XLOC_10087, XLOC_023657, XLOC_060540, XLOC_068691, XLOC_050367, XLOC_043352, XLOC_010372, XLOC_008725 and XLOC_064331.

### 3.8. The qRT-PCR Validation of the Sequencing Data

**Figure 7:**
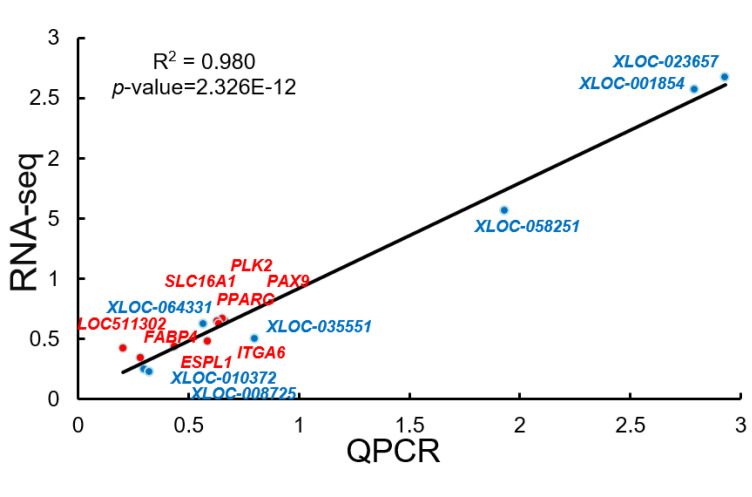
Correlation analysis of RNA-seq and qRT-PCR results.

Correlation analysis of RNA-seq and qRT-PCR results were shown in Figure 7. There were seven DEGs and seven DE lncRNAs that were selected, and the expression of these RNAs were determined by qRT-PCR. Our results showed that the expression of the selected mRNAs and lncRNAs was consistent with the RNA-seq results.

## 4. Discussion

The effects of different NFC/NDF ratio diets on the growth performance of calves were mainly reflected in the change of ADG. Most obviously, the ADG of calves in the H group was significantly higher than that in other groups. Feeding high concentrate diets to calves result in a higher growth performance. High concentrate diets result in higher propionate concentrations in the rumen which results in greater gluconeogenesis and lean tissue deposition; the physiological development of rumen was mainly reflected in the growth of rumen papilla and muscle layer thickness [23]. In the current study, the ruminal papillae of calves fed with the high NFC/NDF ratio diet had a flattened appearance versus a slenderer appearance in the lower concentrate diet. The flattened structure of the rumen papillae would increase available surface area for nutrient absorption and improve calves’ growth performance. The length of the ruminal papilla altered as the proportion of dietary concentrate and nutrient levels changes [24]. The dietary composition determines the pattern of rumen fermentation which was the main factor affecting the rumen development of calves. In particular, an appropriate intake of concentrate could stimulate ruminal papilla growth and development [25]. The high NFC/NDF ratio diet could accelerate rumen development presumably as a result of butyrate generation inducing [26]. Likewise, a certain amount of roughage was also important for maintaining the degree of keratinization of the rumen epithelium [27]. Current results indicated that a high NFC/NDF ratio diet was more conducive to ruminal papillae development in calves.

The application of high-throughput sequencing technology has led to a revolutionary advance in genetic research, allowing researchers to explore the physiological and biochemical processes during growth and development at genomic level. In the current research, 47 differentially expressed lncRNAs, as well as 210 differentially expressed TUCPs and 1304 differentially expressed mRNAs were identified. The newly identified lncRNAs from rumen were shorter in length, fewer in exon number, lower in expression level and less conserved than protein-coding transcripts [28]. Integration of differentially expressed lncRNAs and DEGs found that 47 DE-lncRNAs regulated 860 DEGs. The changes of these genes reflected the prediction that novel lncRNAs play an important role in rumen development.

The lncRNA regulates the expression of adjacent or overlapping protein-coding genes, and the function of lncRNAs might be reflected in their associated protein-coding genes [29]. By co-expression and co-location network analysis, mRNAs with the similar expression pattern as lncRNAs could be found. In order to understand the potential functions of the lncRNAs identified, functional enrichment analyses on the target genes of DE lncRNAs were performed. It was obvious that the functional GO enrichment of these DEGs were mainly related to the process of cell mitosis, which basically covered the whole division stage. Mitosis is the mechanism of self-replication of a single cell, which supports the growth, development and tissue repair of mammals [30]. Entry into mitosis is triggered by the activation of cyclin-dependent kinase 1 (CDK1) [31]. Oscillations in the number and activity of various Cyclin/CDK complexes are critical to cell cycle progression [32]. Therefore, the results of GO enrichment of these DEGs speculated that feeding calves with high and low NFC/NDF ratio diets might affect the number of Cyclin/CDK complex, and then affect the cell division cycle, which resulting in differential development of the ruminal papilla.

The results of KEGG pathway analysis showed that the cGMP-PKG signaling pathway, Wnt signaling pathway and thyroid hormone signaling pathway were significantly enriched. The cGMP-PKG pathway is associated with the growth and apoptosis of cells, of which promoting the transcription of brain-derived neurotrophic factor (BDNF) and stimulating cell proliferation, differentiation and survival [33,34]. Current results suggested that the high fiber provided by low NFC/NDF ratio diet could increase the expression of BDNF through XLOC_039645, increase the intracellular cGMP level [35], activate cGMP-dependent PKG [36], and inhibit the growth and apoptosis of rumen epithelial cells. The Wnt signaling pathway is vital to modulate a myriad of biological processes, including cell fate determination, cell differentiation and cell proliferation [37]. In present study, the high NFC/NDF ratio diet might induce the differentiation of rumen cells and promote the maturation of rumen cells by activating the canonical Wnt signaling pathway. Moreover, thyroid hormone (TH) signaling pathway plays an important role in a wide variety of cellular processes in vertebrates, including cellular proliferation, differentiation and apoptosis [38,39]. The Wnt/β-catenin and bone morphogenetic protein 4 (BMP4) signaling pathways were involved in monitoring continuous self-renewal/diffusion/differentiation procedures during intestinal development [40]. This study speculated that XLOC_032926 down-regulated BMP4, the direct target of TH, thereby enhancing the role of Wnt signaling pathway and inducing cell differentiation [41]. The above results indicated that it was necessary to further target the autonomous metabolism mechanism of rumen cells, through which the TH signal was utilized to promote the self-renewal potential, and it was suggested that the combination of the TH and Wnt signaling pathways to induce cell differentiation might be a promising regulatory approach.

The current work identified three key genes involved in regulating rumen development, including MAOB, DKK2 and PPP1R12A. The MAOB, a key DEG involved by differentially expressed lncRNA, not only could be cis-regulated but also trans-regulated mode with XLOC_068691, which is involved in the tryptophan metabolism pathway. Tryptophan is the precursor of serotonin (5-HT), which was shown to affect cell proliferation, cell migration and cell differentiation [42]. This suggested that the MAOB gene played an important role in the development of the body. The expression of MAOB was down-regulated when the calves were fed with the low-NFC/NDF ratio diet. Studies had reported that the deletion of MAOB in a male patient caused severe developmental delay [43]. This was consistent with the shorter phenotype of the ruminal papilla in L Group calves. The DKK2 was regulated by XLOC_008734 and XLOC_037328, which might promote rumen development by regulating Wnt signaling pathway [44]. As mentioned above, the Wnt signaling pathway is involved in the induction of cell differentiation. The DKK2 binds to and inhibits the Wnt co-receptor LRP6 and was the most critical antagonist of the Wnt signaling pathway [45]. The DKK2-mediated repression of the Wnt/β-catenin pathway is essential to promoting the differentiation of the corneal epithelial progenitor cells into a non-keratinizing stratified epithelium [46]. The PPP1R12A was involved in wound healing, had a positive impact on the migration of keratinocytes and influenced the cell adhesion properties [47]. Interestingly, we found that PPP1R12A was highly expressed in the low NFC/NDF ratio group of calves compared to the high NFC/NDF ratio group of calves. We speculated that a low NFC/NDF ratio with more roughage would cause more mechanical movement in the rumen and increase the friction of the rumen wall. In this state, the body itself adopted a high expression of PPP1R12A to cope with the stimulation brought on by the roughage, because this gene is conducive to the healing of the cutaneous wound. The PPP1R12A accelerated cutaneous wound healing by regulating migration and differentiation of epidermal keratinocytes [48]. In the differentially expressed gene interaction network, PPP1R12A was regulated by XLOC_064331, which might be related to the development of rumen, but the specific mechanism of action needs to be further research.

## 5. Conclusions

The high NFC/NDF ratio diet fed to calves in this experiment promoted enhanced rumen health and development (as determined by size and papillae morphology) which resulted in greater growth performance before 180 days of age. A total of 47 differentially expressed lncRNAs and 1304 differentially expressed mRNAs were identified from rumen tissue among calves fed with different NFC/NDF ratio diets. The target genes of differentially expressed lncRNAs were significantly enriched in pathways closely related to rumen epithelial development including focal adhesion, Wnt signaling pathway, thyroid hormone signaling pathway, regulation of actin cytoskeleton and cGMP-PKG signaling pathway. Some target genes including XLOC_068691, MOAB, XLOC_023657, DKK2, XLOC_064331 and PPP1R12A play important roles in rumen development in calves. These target genes and pathways may be further involved in the absorption and transport of propionate, further improving the growth performance of calves at this stage. The pathways and affected genes may serve as markers for further investigation into the mechanisms regulating dietary and luminal factors impacting rumen development in growing ruminants. The differentially expressed lncRNAs screened were involved in the regulation of rumen development in calves, and the results will provide a theoretical basis for further analyzing the molecular genetic mechanism of dietary factors affecting rumen development in calves.

## Figures and Tables

**Table 1:** Composition and nutrient levels of basal diets.

	Treatment ^(1)^		
Items	H Group	M Group	L Group
Ingredients (% of dry matter)			
Corn	43.62	48.00	30.03
Wheat bran	15.00	0.00	0.00
Soybean meal	2.90	4.30	2.57
Dried distiller’s grains with soluble	15.00	15.00	15.00
Alfafa	20.00	25.00	35.00
Chinese wild rye	0.00	5.00	15.00
Limestone	0.20	0.61	0.21
CaHPO_4_	1.78	0.59	0.69
Premix ^(2)^	1.00	1.00	1.00
NaCl	0.50	0.50	0.50
Total	100.00	100.00	100.00
Nutrient levels (% of dry matter, unless noted)			
Dry matter	91.8	90.5	91.58
Crude protein	16.34	16.42	16.38
Ether extract	3.71	3.54	3.82
Ash	7.57	7.93	7.44
Neutral detergent fiber	34.43	37.14	45.33
Acid detergent fiber	15.34	18.33	25.44
Calcium	1.05	1.08	1.14
Phosphorus	0.45	0.45	0.47
ME/(MJ/kg) ^(3)^	11.20	10.87	9.79
NFC/NDF ^(4)^	1.10	0.94	0.60

^(1)^ H group (NFC/NDF = 1.10). M group (NFC/NDF = 0.94). L group (NFC/NDF = 0.60). ^(2)^ The premix provided the following per kg of the concentrate: VA 15,000 IU, VD 5000 IU, VE 50 mg, Fe 90 mg, Cu 12.5 mg, Mn 60 mg, Zn 100 mg, Se 0.3 mg, I 1.0 mg, Co 0.5 mg. ^(3)^ ME was a calculated value, and the other nutrient levels were measured values. ^(4)^ NFC (%) =100 − (NDF + CP + EE + Ash). NFC: non-fibrous carbohydrate; NDF: neutral detergent fiber.

**Table 2:** Primer information used for qPCR.

Accession NO.	Gene Symbol	Primer Sequence (5′ to 3′)	Annealing Temperature (°C)	Product Size
NM_174314.2	*FABP4*	F: AGTTTGAATGGGGGTGTGGT	58	199 bp
		R: CGAGTTTTCTCTTTATGGTGGT		
NM_001037319.1	*SLC16A1*	F: ATGCCACCACCAGTGAAGTG	60	216 bp
		R: GCCCAAGACCTCCAATGACT		
NM_001192369.1	*PAX9*	F: AGTATTCGTGAACGGGAGGC	60	164 bp
		R: GCAAGATCGAGCCTGTCTCA		
NM_001192245.1	*PLK2*	F: TCTCCATCACAAGCACGTCG	60	273 bp
		R: GCCAAACCAAAGTCCCCAAC		
NM_001109981.2	*ITGA6*	F: CGAAGCAGGAATCCCGAGAC	60	296 bp
		R: TCCACCAACTTCATAAGGCCC		
NM_181024.2	*PPARG*	F: CAAGAGCTGACCCGATGGTT	60	193 bp
		R: CCTGACGCTTTATCCCCACA		
NM_001045949.2	*ESPL1*	F: TGAAGCCAGGCACCTATCC	58	195 bp
		R: CCATCTTGACCCGAACCCA		

**Table 3:** Effects of diets with different NFC/NDF on the growth performance of calves.

Items	Treatment ^1^			SEM ^2^	*p*-Value
	H Group	M Group	L Group		
Initial body weight/kg	95.04	93.82	93.23	9.27	0.481
Final body weight/kg	190.88	183.43	171.27	2.96	0.964
Body weight gain/kg	92.73 ^a^	77.07 ^b^	70.87 ^b^	3.12	0.009
Average daily gain/kg	1.14 ^a^	1.00 ^b^	0.93 ^b^	0.03	0.008
Dry matter intake/(kg/d)	4.09	3.81	3.85	0.11	0.605
Feed to gain ratio	3.65	3.81	4.15	0.09	0.169

^a^^,^^b^ Means within a row with different superscripts differ (*p* < 0.05). ^1^ The calves in H group were fed diet with H, NFC/NDF = 1.10. M, NFC/NDF = 0.94. L, NFC/NDF = 0.60. **^2^** SEM=Standard error of mean.

**Table 4:** Effects of different diets on the fermentation parameters and morphology of rumen.

Items	Treatments ^1^			SEM ^2^	*p*-Value
	H Group	M Group	L Group		
pH	6.81	7.00	6.83	0.06	0.419
Acetate	53.43 ^b^	59.92 ^a^	60.71 ^a^	1.24	0.026
Propionate (mmol/L)	25.77 ^a^	21.66 ^ab^	18.96 ^b^	1.01	0.014
Butyrate (mmol/L)	13.36	12.26	12.76	0.80	0.875
Papillae length (mm)	0.75 ^b^	0.92 ^b^	1.11 ^a^	0.08	0.023
Papillae width (mm)	0.38 ^a^	0.34 ^a^	0.24 ^b^	0.01	<0.001
Muscle layer thickness (mm)	1.21	1.23	1.35	0.04	0.144

^a,b^ Means within a row with different superscripts differ (*p* < 0.05). ^1^ The calves in H group were fed diet with H, NFC/NDF = 1.10. M, NFC/NDF = 0.94. L, NFC/NDF = 0.60. ^2^ SEM = Standard error of mean.

**Table 5:** Summary of draft reads of six libraries by RNA-sequencing.

Items	H-1 ^1^	H-2 ^2^	H-3 ^3^	L-1 ^4^	L-2 ^5^	L-3 ^6^
Raw reads	129,008,240	97,533,606	113,893,888	84,213,254	89,185,784	99,002,628
Clean reads	126,165,758	95,293,260	111,116,586	81,352,198	87,101,750	96,764,402
Percentage (%)	97.80	97.70	97.56	96.60	97.66	97.74
Mapped reads	104,668,662	80,985,576	93,383,745	67,890,749	72,068,883	82,345,847
Mapping rate (%)	82.96	84.99	84.04	83.45	82.74	85.10
Q30 (%)	93.64	93.23	92.68	91.93	93.83	93.33
Spliced reads (%)	20.59	18.07	21.56	22.23	20.8	22.1
Non-spliced reads (%)	59.65	64.54	59.59	57.47	58.57	60.21

^1–3^ H-1, H-2 and H-3 were three libraries established of H Group (NFC/NDF = 1.10) by RNA-sequencing; ^4–6^ L-1, L-2 and L-3 were three libraries established of L Group (NFC/NDF = 0.60) by RNA-sequencing.

## Data Availability

The data presented in this study are available from the corresponding author on reasonable request.
